# The Biological Deep Sea Hydrothermal Vent as a Model to Study Carbon Dioxide Capturing Enzymes

**DOI:** 10.3390/md9050719

**Published:** 2011-04-28

**Authors:** Zoran Minic, Premila D. Thongbam

**Affiliations:** 1 Department of Chemistry, University of Saskatchewan, 110 Science Place, Saskatoon, SK S7N 5C9, Canada; 2 Biochemistry Laboratory, ICAR Research Complex for North Eastern Hill Region, Umiam, Meghalaya 793103, India; E-Mail: pthongbam2000@yahoo.co.in

**Keywords:** deep sea hydrothermal vents, carbon dioxide, fixation, assimilation, capturing enzyme

## Abstract

Deep sea hydrothermal vents are located along the mid-ocean ridge system, near volcanically active areas, where tectonic plates are moving away from each other. Sea water penetrates the fissures of the volcanic bed and is heated by magma. This heated sea water rises to the surface dissolving large amounts of minerals which provide a source of energy and nutrients to chemoautotrophic organisms. Although this environment is characterized by extreme conditions (high temperature, high pressure, chemical toxicity, acidic pH and absence of photosynthesis) a diversity of microorganisms and many animal species are specially adapted to this hostile environment. These organisms have developed a very efficient metabolism for the assimilation of inorganic CO_2_ from the external environment. In order to develop technology for the capture of carbon dioxide to reduce greenhouse gases in the atmosphere, enzymes involved in CO_2_ fixation and assimilation might be very useful. This review describes some current research concerning CO_2_ fixation and assimilation in the deep sea environment and possible biotechnological application of enzymes for carbon dioxide capture.

## 1. Introduction

Some organisms are capable of synthesizing complex organic molecules from simpler inorganic compounds such as carbon dioxide (CO_2_), minerals and water. On Earth, photosynthetic organisms are able to manufacture complex organic molecules from simple inorganic compounds using the energy from sunlight. These organisms include plants, green algae, some protists (such as phytoplankton), and some bacteria (such as cyanobacteria) [1,2]. Photosynthetic organisms can create their own food and these are called autotrophs, meaning “self-feeding”. Autotrophs also are referred to as primary producers. It has been estimated that their total net primary production on Earth exceeds 104.9 petagrams of carbon per year, and that they play a crucial role in the global carbon cycle [1]. It is important to note that roughly half of this productivity occurs in the oceans and is mainly performed by microscopic organisms called phytoplankton. Although the concentration of carbon dioxide in the atmosphere is low, about 0.039%, this gas is indispensable for terrestrial photosynthesis. However, in some environments, primary production happens though a process called chemosynthesis [3]. Chemosynthetic organisms are autotrophs capable of obtaining energy to make their organic food by oxidizing high-energy inorganic compounds (hydrogen gas, ammonia, nitrates, and sulfides). On Earth, chemosynthetic ecosystems include hot vents, cold seeps, mud volcanoes and sulfidic brine pools. Therefore, autotrophs of different types can produce energy either through photosynthesis or chemosynthesis.

In 1977, marine scientists discovered ecosystems based on chemosynthesis at a depth of 2.5 km around a hot spring on the Galapagos volcanic Rift (spreading ridge) off the coast of Ecuador [4,5]. Since then, several hydrothermal vents rich in living organisms have been discovered and explored along the volcanic ridges in the Atlantic and Pacific oceans. The environments of these hydrothermal vents are considered extreme with unique physical and chemical properties such as elevated pressure (up to 420 atm), high and rapidly changing temperature (from 2–4 °C to 400 °C), acidic pH, toxic heavy metals, hydrogen sulfide and complete absence of light [6,7]. The origin of deep hydrothermal vents is continental drift. The lithosphere is divided into seven major and several minor plates all of which are moving relative to each other, creating cracks and crevices in the ocean floor [8]. These plates are separated by ridges divided into multiple segments separated by fracture zones. The rate of expansion of dorsal segments varies from 1 to 280 mm per year. The fracture zone is characterized by strong volcanic activity. Seawater seeps into these openings and is heated by the molten rock, or magma, which can reach very high temperatures (up to 400 °C) and then this hydrothermal fluid of heated sea water rises back to the surface dissolving large amounts of minerals which provide a source of energy and nutrients to chemoautotrophic organisms [9–17]. Numerous living organisms have been discovered in these hostile environments including microorganisms (Eubacteria and Archaea) and pluricellular organisms such as shrimps, clams and giant mussels, giant tubeworms, crabs and fishes [18]. These organisms have developed different strategies to ensure their adaptation to these extreme environments. In the total absence of photosynthesis, the food chain is based on the primary production of energy and organic molecules by chemolithoautotrophic bacteria, free or more or less associated to some of these organisms such as tubeworms, mussels and clams [6,7,17]. The environment around hydrothermal vents, where different populations of bacteria live, is characterized by large temperature variation. Some microorganisms thrive at the low temperature of 1–4 °C prevailing in the deep sea (cold-adapted psychrophilic bacteria), others, mesophiles, at moderate temperatures (10–60 °C) and finally, some strains, called thermophiles or hyperthermophiles, thrive at around 60 °C and 100 °C, respectively [17]. For example, the archaea *Pyrolobus fumarii* can grow at 113 °C with an optimal temperature of growth at 106 °C [19]. In contrast to photosynthetic organisms that use solar energy, carbon dioxide, nutrients and water to produce organic materials and thereby biomass, chemiolithoautotrophic bacteria in deep sea hydrothermal vents are able to extract chemical energy starting from the oxidation of reduced mineral compounds present in their habitat. Then, this energy is used to synthesize complex organic molecules from simpler inorganic compounds such as carbon dioxide, nitrate, ammonium and other minerals. The synthesized small organic molecules are available to a number of animal species, which live in an obligate symbiosis with these chemosynthetic bacteria (clams, mussels, gastropods and vestimentiferan tubeworms). The hydrogen sulfide (H_2_S) and heavy metals (Pb, Cd, Hg, Zn, *etc.*) which are present in high concentrations in the hydrothermal vents are toxic for living organisms. The organisms of this ecosystem developed efficient mechanisms of defense to protect themselves from these toxic materials [17]. For example, they use unusual enzymes capable of resisting high temperatures and pressures. In addition, the adaptation of these microorganisms implies that there must be many others modifications of their biochemical components such as proteins, membranes and nucleic acids, as well as other physiological modes of adaptation.

Overall, autotrophic organisms (photosynthetic or chemoautotrophic) can use different sources of energy such as light or reduced minerals to synthesize complex organic molecules but possess the common characteristic of being able to incorporate carbon from CO_2_ into organic compounds. This aspect of carbon fixation has been mentioned in some excellent reviews [20–24]. In this review, we summarize current knowledge about enzymes that are involved in carbon dioxide fixation and assimilation, such as carbonic anhydrase, by organisms associated with deep sea hydrothermal vents. Because of the fact that this environment is characterized not only by diversity in physical and chemical factors but also by microbial and animal biodiversity, suggests that enzymes from these organisms might be of interest in different biotechnological strategies regardless of carbon dioxide capturing.

## 2. Carbon Dioxide in the Environments of Marine Hydrothermal Vents

The organisms in hydrothermal vents, are exposed to wide variations in dissolved inorganic carbon species (DIC) = CO_2_ + HCO_3_^−^ + CO_3_^2–^ = 2 to 7 mM; pH = 5 to 8 and bottom water (DIC = 2 mM; pH = 8) [25]. In this environment with constantly changing pH and DIC, the CO_2_ concentration oscillates between 20 μM and 1 mM [25–28]. Although these habitats are characterized with oscillations in the availability of the inorganic nutrients necessary for chemolithoautotrophic growth, many organisms have adapted to this fluctuation in the concentration of dissolved inorganic carbon. One of the strategies of adaptation, in the presence of low concentrations of dissolved inorganic carbon, is cellular ability to use both HCO_3_^−^ and CO_2_ [29]. In contrast to plants that are able to fix CO_2_ via a Calvin Benson cycle, the organisms of hydrothermal vents use multiple mechanisms for fixation of CO_2_ such as Calvin-Benson cycle, reductive tricarboxylic acid cycle (reductive TCA cycle), acetyl-CoA pathway, dicarboxylate/4-hydroxybutyrate cycle and 3-hydroxypropionate/4-hydroxybutyrate cycle.

## 3. Fixation and Assimilation of Carbon

**Figure 1.**
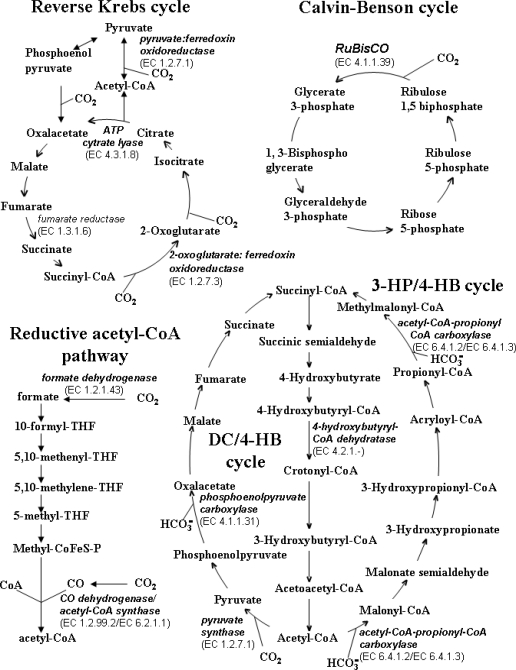
Pathways of autotrophic CO_2_ fixation: reverse Krebs cycle (reductive TCA cycle), Calvin-Benson cycle, acetyl CoA-pathway, dicarboxylate/4-hydroxybutyrate and 3-hydroxypropionate/4-hydroxybutyrate cycle. The reactions catalyzed by key enzymes are shown in italics. Abbreviations: THF, tetrahydrofolate; CoA, coenzyme A; CoFe/S-P, carrinoid-iron sulfur protein; RuBisCO, Ribulose-1,5-bisphosphate carboxylase/oxygenase.

Based on numerous reports, organisms of deep sea hydrothermal vents use several metabolic pathways for CO_2_ fixation. These include the Calvin-Benson cycle, reductive TCA cycle, 3-hydroxypropionate bicycle, acetyl CoA-pathway, dicarboxylate/4-hydroxybutyrate cycle and 3-hydroxypropionate/4-hydroxybutyrate cycle [20–24] (Figure 1).

As shown, several enzymes are associated with CO_2_ fixation such as adenosine triphosphate (ATP) citrate lyase, 2-oxoglutarate: ferredoxin oxidoreductase and fumarate reductase, ribulose-1,5-bisphosphate carboxylase, acetyl-CoA carboxylase, propionyl-CoA carboxylase, formate dehydrogenase, CO dehydrogenase/acetyl CoA synthase, 4-hydroxybutyryl-CoA dehydratase and acetyl-CoA-propionyl-CoA-carboxylase. In addition, organisms of deep sea vents possess different forms of carbonic anhydrase important for assimilation of CO_2_ [30–34].

### 3.1. Calvin-Benson Cycle

In the Calvin-Benson cycle, ribulose 1,5-bisphosphate carboxylase/oxygenase (RuBisCO, EC 4.1.1.39) catalyzes the addition of molecular CO_2_ to ribulose 1,5-bisphosphate generating two molecules of 3-phosphoglycerate (3-PGA) [21]. This reaction (carboxylase reaction) is the essential step in the transformation of atmospheric inorganic carbon to organic carbon in our biosphere, and 3-PGA plays a key role in the synthesis of all organic cell materials. This is the most abundant enzyme on earth and plays a crucial role in CO_2_ fixation.

The first demonstration of the existence of enzymes of the Calvin-Benson cycle associated with hydrothermal vents was demonstrated by Felbeck [12]. High activities of ribulose-1,5-bisphosphate carboxylase/oxygenase and ribulose 5-phosphate kinase, enzymes of the Calvin-Benson cycle of CO_2_ fixation, have been detected in the trophosome of *Riftia pachiptila. Riftia pachyptila* is the giant tubeworm (Siboglinidae annelide polychaete) present in the East Pacific Ridge. This animal is devoid of a digestive tract and lives in an intimate symbiosis with a sulfur-oxidizing chemoautotrophic bacterium that is localized in the cells of a richly vascularized organ of the worm: The trophosome [16,17,35]. It is interesting to note that the detected activities of two enzymes, ribulosebisphosphate carboxylase and ribulose 5-phosphate kinase, are present at high levels in trophosome (the organ housing the symbiotic bacteria), but are absent in muscle [12]. In addition, enzymatic activities of rhodanese, APSreductase, and ATP-sulfurylase were positive and these enzymes are involved in the synthesis of adenosine triphosphate using energy contained in sulfur compounds such as hydrogen sulfide. These results lead to the conclusion that the *R. pachyptila* symbiont is capable of generating ATP by way of sulfide oxidation and energy from ATP could be used to fix CO_2_.

In addition to the *Riftia pachyptila* symbiont, other deep-sea invertebrate symbionts that utilize the Calvin cycle for CO_2_ fixation have since been characterized [12,36–40].

Studies of microbial community at different hydrothermal vent sites have revealed that these microorganisms fix carbon dioxide using the Calvin-Benson cycle [41,42]. Several investigations reported the presence of RuBisCO in archaeal, bacterial and eukaryal species in different hydrothermal vents [43–46]. It is important to note that over the past 3.5 billion years, with decreasing CO_2_ and increasing O_2_ in the atmosphere, RuBisCO has also evolved into multiple enzymatic forms having a range of functional properties [47]. Data obtained from structural and sequence homology of the enzymes using cyanobacterial RuBisCO provided information that the cyanobacteria are the origin of plant chloroplasts [48,49]. There are four forms of RuBisCO. Forms I, II and III catalyse the same reactions, carboxylation or oxygenation of ribulose 1,5-bisphosphate. Form I is comprised of small and large subunits [44,50], whereas form II possesses two identical large subunits [44,51–54]. Form III and IV consist of the large subunits only. Form IV constitutes the RuBisCO-like proteins (RLPs) because this protein does not catalyse RuBisCO activity.

RuBisCO form I has been classified into four types, IA–ID, based on the amino acid homology of the large subunit genes (*cbbL*) [55]. Previous studies showed that the bacterial forms IA/IC and II occur in deep sea hydrothermal vents. Form IA is present in Proteobacteria [44,56,57] and in the symbionts of several deep-sea hydrothermal vents such as mollusks and some Pogonophora species [58–61]. Form IB has only been identified from a vent plume at a Mid-Okinawa trough hydrothermal vent. Recently, studies of three Western Pacific arc hydrothermal systems revealed both archaeal forms IC and ID, in addition to form IA and form II [43] from the Pika/Suiyo and Suiyo samples, respectively. Form II has been identified in free-living autotrophic microbial communities at only two hydrothermal vent sites [44,57]. In addition, form II was detected as the predominant form in cold seeps, symbionts of tubeworms and some deep-sea clams [62,63]. The crystal structure and biochemical studies of RuBisCO (Tk-Rubisco) from the hyperthermophilic archaeon *Thermococcus kodakaraensis* KOD1 indicated that its structure is distinct from form I and II RuBisCO [64]. This Tk-Rubisco is classified as a novel form III. Form IV has been found in *Archaeoglobus fulgidus*, a hyperthermophilic sulfate-reducing Archaeon, from marine hydrothermal systems [65].

### 3.2. Krebs Reverse Cycle (Reductive Tricarboxylic Acid Cycle)

The reverse tricarboxylic acid (rTCA) cycle fixes CO_2_ and leads to the synthesis of acetyl coenzyme A, which is carboxylated to pyruvate. The three key enzymes essential to run the rTCA cycle include ATP citrate lyase, 2-oxoglutarate: ferredoxin oxidoreductase and fumarate reductase (Figure 1).

Pioneer work on the presence of the rTCA cycle in deep-sea hydrothermal vent microbial communities has been obtained from expression studies of the episymbiotic community associated with the vent polychaete *Alvinella pompejana* [57,66,67]. This deep-sea polychaete *Alvinella pompejana* colonizes tubes on the sides of black smoker chimneys along the East Pacific Rise. The two key genes in the rTCA cycle, the ATP citrate lyase and 2-oxoglutarate: ferredoxin oxidoreductase, were utilized to demonstrate the abundance of these genes in the episymbiont community [66] and in diverse vent samples [57]. The overall results of these analyses demonstrated: (i) the presence and expression of key rTCA cycle genes in at least two groups of free-living microorganisms from deep-sea hydrothermal vents; and (ii) the majority of autotrophs that utilize the reverse tricarboxylic acid cycle are members of the epsilon subdivision of Proteobacteria. Concerning the best studied organism in deep sea hydrothermal vents, the giant tubeworm *Riftia pachyptila*, its chemoautotrophic gamma proteobacterial endosymbionts fix carbon via the Calvin cycle [12,13,16,17,35]. In addition, recent proteomic analyses revealed the presence of enzymes involved in the reverse TCA cycle [68]. These results indicated that *Riftia* uses both the reverse TCA pathway and the Calvin cycle for fixation of CO_2_.

Several studies of the occurrence of the rTCA cycle in microbial communities of deep-sea hydrothermal habitats have been carried out. These studies demonstrated that pure cultures of Epsilonproteobacteria and Aquificae, which include representatives of hydrothermal vent bacteria, fix carbon dioxide via the rTCA cycle [69–76]. Comparative analyses of the ATP citrate lyase encoding genes from natural microbial communities of three deep-sea hydrothermal vent systems located on the Mid-Atlantic Ridge (MAR; Rainbow, Logatchev and Broken Spur) suggested that Epsilonproteobacteria were the dominant primary producers using the reverse TCA cycle (rTCA) at Rainbow, while Aquificales of the genera Desulfurobacterium and Persephonella were prevalent in the Broken Spur chimney [76]. Several studies indicate that the majority of bacteria associated with deep-sea hydrothermal chimneys are members of the Epsilonproteobacteria [73,77–81]. The habitat of these bacteria are located in the zone where hydrothermal fluids mix with ambient seawater giving temperatures in the range from 20 to 60 °C and characterized by microaerobic to anaerobic conditions [71]. Since the energy in these environments is limited, it is more favorable to use a carbon fixation pathway other than the Calvin cycle, which requires nine ATP molecules to synthesize one triose phosphate molecule compared to five ATP for the reductive TCA cycle [82]. The fact that the Epsilonproteobacteria at hydrothermal vent sites express enzymes of rTCA, suggests that the rTCA cycle could play a more important role in chemoautotrophic production by free-living microorganisms at hydrothermal vents than the Calvin cycle.

### 3.3. 3-Hydroxypropionate Bicycle

The key enzymes for the 3-hydroxypropionate (3-HP) bicycle are two carboxylases: acetyl-CoA/propionyl-CoA carboxylase. The 3-hydroxypropionate bicycle appears to be restricted to Chloroflexaceae [24,83–85]. Consequently, this cycle may not be present in deep sea vents.

### 3.4. Reductive Acetyl-CoA Pathway

The reductive acetyl-CoA pathway (Figure 1) results in the fixation of two molecules of CO_2_ to form acetyl-CoA. One molecule of CO_2_ is consecutively reduced to a cofactor-bound methyl residue, and another CO_2_ molecule is reduced to an enzyme-bound carbonyl residue. The key enzyme of the reductive acetyl-CoA pathway is a bifunctional metalloenzyme belonging to the class of oxidoreductases, CO dehydrogenase/acetyl-CoA synthase, which catalyzes the reversible reduction of CO_2_ to CO and the assembly of acetyl-CoA from CO, Coenzyme A and a methyl moiety derived from the corrinoid iron-sulfur protein [86,87]. The reduction of CO_2_ to the methyl group is accomplished by a series of enzymes, most of which are also unique for this pathway [88,89]. The formate dehydrogenase in this pathway is important because it “reductively fixes” CO_2_ to formate.

At deep-sea hydrothermal vents, the majority of methanogenic microorganisms use the reductive acetyl-CoA pathway for carbon fixation. These methanogens include *Methanococcales* (e.g., *Methanocaldococcus* and *Methanothermococcus*) and *Methanopyrales* (*i.e.*, Methanopyrus) [20] and are identified within mid-ocean ridge systems, backarc basin systems [90,91] and also in ridge flank systems [92].

### 3.5. Dicarboxylate/4-Hydroxybutyrate Cycle

The complete metabolic cycle of dicarboxylate/4-hydroxybutyrate (DC/4-HB) was elucidated recently by Huber *et al.* [93] (Figure 1). The key enzyme of the DC/4-HB cycle is 4-hydroxybutyryl-CoA dehydratase [94,95]. This pathway occurs in the thermophilic crenarchaeon *Ignicoccus hospitalis* (Desulfurococcales), but it is also present in other Crenarchaeota such as Thermoproteales and Desulfurococcales [23,93,96]. *I. hospitalis* is a chemolithoautotrophic, sulfur reducer and hyperthermophilic crenarchaeon which was isolated from a submarine hydrothermal system at the Kolbeinsey Ridge, to the north of Iceland [97]. Microbial analyses at several deep sea hydrothermal vent sites showed the abundance of archaeal domains Crenarchaeota, including the group closely associated with *Ignicoccus* [98–100]. This evidence suggests that the fixation of CO_2_ might occur through dicarboxylate/4-hydroxybutyrate cycle at deep sea hydrothermal vents.

### 3.6. 3-Hydroxypropionate/4-Hydroxybutyrate Cycle

The 3-HP/4-HB cycle functions in the autotrophic thermoacidophilic members of the crenarchaeal order Sulfolobales [23,101–103] (Figure 1). The key enzyme of the 3-HP/4-HB cycle, 4-hydroxybutyryl-CoA dehydratase, was identified in the genome of marine group 1 Crenarchaeota [104] and in the Global Ocean Sampling database [101,105,106]. A recent study showed that anaerobic ammonium-oxidizing bacteria and archaea are present and active in hydrothermal vent areas [107]. Therefore, it appears that the 3-HP/4-hydroxybutyrate is potentially an important carbon fixation pathway in deep-sea hydrothermal vents.

### 3.7. Carbonic Anhydrase

In general, the inorganic carbon acquisition and fixation by marine species depend upon changes in CO_2_ and/or HCO_3_^−^ concentrations [108–110]. The carbonic anhydrases (CA) are enzymes that facilitate DIC uptake and fixation [33,30]. These enzymes catalyze the reversible hydration of CO_2_ to form bicarbonate [CO_2_ + H_2_O ↔ HCO_3_^−^ + H^+^]. In typical physiological solutions, CO_2_(aq) is in equilibrium with HCO_3_^−^ (aq). The HCO_3_^−^ is negatively charged and poorly soluble in lipids, while CO_2_ is highly soluble in lipids. Therefore, CO_2_ can freely diffuse out of the cell but HCO_3_^−^ must be transported across the cell membrane. However, some microorganisms such as cyanobacteria possess a transport system specialized for CO_2_ uptake by converting CO_2_ to HCO_3_^−^ [111,112]. In addition, above pH 6.3, the equilibrium between these two species shifts toward HCO_3_^−^ and thus poses problems in the maintenance of intracellular CO_2_ [30]. For trapping CO_2_ in the cell, HCO_3_^−^ is enzymatically converted to CO_2_ and in this manner it facilitates its transport into the cell. In the cell, the RuBisCO enzyme is restricted to using CO_2_ for its function and the efficiency of CO_2_ fixation is enhanced by a specialized carbonic anhydrase that catalyzes dehydration of the cytoplasmic bicarbonate and ensures saturation of RuBisCO with its substrate [33,113].

Investigations of CAs in the model organism *Riftia pachyptila*, revealed the presence of several isoforms of this enzyme [31,32,34]. Inorganic carbon (CO_2_) is first acquired from the environment by diffusion across the plume, a branchial organ [26]. At the environment-branchial plume interface CO_2_ is converted to HCO_3_^−^ [114,115] and is transported to trophosome cells mainly in the form of bicarbonate. HCO_3_^−^ is converted to CO_2_ at the body fluid-bacteriocyte interface [31,32,34] and fixed by the bacterial symbionts enzyme RuBisCO form II [21].

In prokaryotes there are three phylogenetically distinct classes of CAs: α, β and γ. Interestingly all three of these classes of CA are present in the *Thiomicrospira crunogena*, a deep-sea hydrothermal vent sulfur-oxidizing chemolithoautotroph that lives in a spatially and thermally heterogeneous environment [33,116]. When the corresponding genes have been expressed in *Escherichia coli*, CA activity was detected for α-CA and β-CA, but not for the γ-CA-like protein [33].

As mentioned above, two general roles have been suggested for the known carbonic anhydrases; the transport of CO_2_ or HCO_3_^−^ and provision of these substrates to cells [30]. Therefore, in addition to CO_2_ transport, the CAs can provide also HCO_3_^−^ for various enzymes that can assimilate carbon. For example, the first enzymes, carbamylphosphate synthetase, in the arginine biosynthetic pathway and the pyrimidine *de novo* pathway use the inorganic HCO_3_^−^ to initiate the biosynthesis [117,118]. The existence of these enzymes has been studied in all the tissues of *Riftia*. The results indicate that the first three enzymes of the *de novo* pyrimidine nucleotide pathway, carbamylphosphate synthetase, aspartate transcarbamylase and dihydroorotase are present only in the trophosome, the symbiont-harboring tissue [117]. Concerning the arginine biosynthetic pathway, it appears that the ammonium dependent carbamylphosphate synthetase is present in all the body parts of *R. pachyptila* as well as in the bacterial symbiont [118]. The unusual distribution of the enzymes of the *de novo* pyrimidine nucleotide pathway in all the tissues of *R. pachyptila* indicates that the metabolic relationship between *R. pachyptila* and its endosymbiont is clearly essential for the survival of both organisms.

## 4. Biotechnological Application

Organisms that live in the environment of deep sea hydrothermal vents characterized by extreme physico-chemical conditions of temperature, pressure, pH and high concentrations of toxic heavy metals represent one of the most important sources for the development of new biotechnological applications. The biotope of hydrothermal vents harbors various and complex microbial communities adapted to different environmental conditions with unique features and characteristics and consequently these organisms could be used in biotechnology. Concerning carbon dioxide fixation and assimilation, the environment of deep sea hydrothermal vents can provide sources of unique enzymes, genes and metabolic processes important for the development of technologies related to industrial processes for reduction of atmospheric CO_2_, biofuels production, materials and chemical synthesis [17,119–122].

Carbon dioxide is the gas that is the major contributor to the green house effect and as such is largely responsible for global warming [123–125]. This gas has been extensively released during the past 100–150 years into the atmosphere due to human activities. Over the past 150 years atmospheric CO_2_ concentrations have increased approximately by 30% [126]. To overcome the effects of global warming there is an urgent need to reduce the atmospheric CO_2_ content. Biotechnological methods have been used to reduce the atmospheric CO_2_ content at two levels; the biological fixation using microorganisms, and the capture of carbon dioxide via enzyme (carbonic anhydrase).

Some microalgae like Cyanophyceae (blue-green algae), Chlorophyceae (green algae), Bacillariophyceae (including diatoms) and Chrysophyceae (including golden algae) are known to be very efficient in utilizing atmospheric CO_2_ via photosynthesis [127,128]. Using genetic engineering and technology, new strains of these microalgae have been developed that can tolerate high concentrations of CO_2_ [127]. In addition, current technologies are being employed to examine the possibility of coupling wastewater treatment with microalgal growth for eventual production of biofuels [127]. Recently, a cyanobacterium, *Synechococcus elongatus* PCC7942 has been genetically engineered to produce isobutyraldehyde and isobutanol directly from CO_2_, increasing productivity by overexpression of ribulose 1,5-bisphosphate carboxylase/oxygenase (RuBisCO) [129]. Isobutyraldehyde is a precursor for the synthesis of other chemicals, and isobutanol can be used as a biofuel. However, a bioreactor that is able to achieve maximum productivity and maximum energy efficiency under a given set of operational costs is not yet fabricated [127]. A major problem with these reactors is related to low efficiency of carbon fixation using the Calvin cycle native to microalgae [130]. In order to develop a new reactor for enhanced microalgal CO_2_ fixation, it is necessary to increase the efficiency of the Calvin cycle. Genetic manipulation of RuBisCO might help to develop a new biotechnological system for large-scale carbon dioxide capture. In addition to the Calvin cycle, other CO_2_ fixation pathways or carboxylase enzymes could be used. These engineering alternatives for CO_2_ fixation strategies might be advantageous as they may avoid the regulatory constraints and substrate limitations of native pathways [130]. Moreover, besides microalgae other microbes, *i.e.*, bacteria and archaea, can also contribute to biofuel production and reduction of global warming [131]. For example, various types of bacteria that use energy obtained from chemical oxidation under dark conditions can be efficient in CO_2_ fixation and can reduce CO_2_ to fuel. These bacteria possess the genes that encode the key enzymes of ethanol biosynthesis from pyruvate. Several studies have showed that CO_2_ may be converted to ethanol by Rhodobacter species under anoxygenic conditions in the light or under dark aerobic growth conditions [132–134]. Therefore, microbes from hydrothermal deep sea vents that can fix CO_2_ into biomass could be of interest for development of the technologies for the production of biofuel as well as other compounds.

Carbonic anhydrases (CAs), the enzymes that catalyze the conversion of CO_2_ to bicarbonate and the selective conversion of CO_2_ to a liquid phase, can separate the CO_2_ from other gases. Therefore, as a potential catalyst, CA could be used in capture of CO_2_ from combustion fuel gas streams [135,136]. Different laboratory-scale reactors have been developed to evaluate the capture of carbon dioxide from a gas into a liquid. The capture efficiencies could be enhanced by adding base (e.g., sodium hydroxide) to form bicarbonate or carbonate, which could be further transformed into insoluble CaCO_3_ by adding precipitating cations, like Ca^2+^ [137]. CaCO_3_ is a thermodynamically stable mineral found in all parts of the world, and is the main component of marine shells, snails, pearls, and eggshells. Sharma *et al.* [138] screened diverse groups of bacteria and found the best activity for CO_2_ conversion was obtained with a 29 kDa CA extracted from *Enterobacter taylorae*. Bhattacharya *et al.* [139] have developed a spray reactor coated with immobilized CA for CO_2_ capture and storage. They obtained a decrease in CO_2_ of almost 70%, and observed stability of CA at 40 °C. Novozymes Inc has a patent application for the cloning and purification of CA for CO_2_ storage [140]. The cloning of CA from *Methanosarcina thermophila* (Archaea) was performed using the bacterium *Bacillus halodurans*, and expressed enzymes were then purified by chromatography. Carbon Sciences Inc. has developed a method for synthetic precipitation of calcium carbonate (PCC) that can be used for various applications, e.g., paper, medicine and plastics production, and in a technology to transform CO_2_ emissions into the basic fuel building blocks required to produce gasoline, diesel, and jet fuel and other fuels. CO_2_ Solution Inc. has developed a method by which CO_2_ emissions from cement factories can be captured and converted into bicarbonate ions. These ions are then used to produce limestone, a raw material that can be reintroduced into the cement manufacturing process [141]. However, existing CAs are expensive due to high manufacturing costs, low activity and stability. The majority of enzymes exhibit very low, or no, activity when the temperature exceeds 50 °C [134]. Most industrial processes to eliminate CO_2_ occur at elevated temperatures, and immobilization techniques to retain biocatalyst activity will need to be performed at relatively higher temperatures [142]. In the environments of deep sea hydrothermal vents, many microorganisms have adapted to high temperatures, toxic substances such as H_2_S and heavy metals. For these reason, biomolecules from these organisms might be of great value in different biotechnological strategies [17]. Therefore, the exploration of carbonic anhydrases for carbon capture from these environments could be attractive for use in new biotechnological applications.

## 5. Conclusions

Deep sea hydrothermal vents are isolated habitats that contain many unique organisms of the three domains of life; archaea, bacteria and eukarya. Most microbial communities in these habitats have the capability to fix inorganic carbon dioxide. Five CO_2_ fixation pathways have been documented as important in hydrothermal habitats; the Calvin-Benson cycle, reductive tricarboxylic acid cycle, reductive acetyl-CoA pathway, dicarboxylate/4-hydroxybutyrate cycle and 3-hydroxypropionate/4-hydroxybutyrate cycle. Four different forms of RuBisCO, designated as I, II, III and IV, operate in different microbial communities associated with deep sea hydrothermal vents. The rTCA cycle is found in the Epsilonproteobacteria and Aquificales and the reductive acetyl-CoA pathway in the methanogens microorganisms. It appears that the 3-HP/4-hydroxybutyrate is potentially an important carbon fixation pathway for archaeal communities in deep-sea hydrothermal vent environments. In addition to these pathways for the direct fixation of carbon dioxide, carbonic anhydrase catalyzes the interconversion of CO_2_ and HCO_3_^−^, and facilitates inorganic carbon dioxide uptake, fixation and assimilation. The bicarbonate formed by CA is an essential growth factor for microorganisms and is a metabolic precursor for many other compounds.

Human activities have significantly increased the atmospheric carbon dioxide concentration and this is an important cause of global warming. Therefore, it is of interest to find technologies for carbon dioxide capture. These technologies, combined with other efforts, could help stabilize greenhouse gas concentrations in the atmosphere and mitigate climate change. Biological CO_2_ fixation has attracted much attention as an alternative strategy. It can be done by plants and by photosynthetic and chemosynthetic microorganisms. These biological technologies could also be attractive for production of biofuels or other industrial products. A variety of technological solutions have been proposed for CO_2_ sequestration systems. In addition, a number of technologies are currently employed or under development to separate carbon dioxide from mixed byproduct streams of large stationary anthropogenic sources. Therefore, a variety of reactors containing an enzyme such as carbonic anhydrase have been designed to extract CO_2_ from mixed gas.

In order to develop and improve new technologies, it is important to search and explore enzymes from different sources. The organisms of deep sea hydrothermal vents are well adapted to fix carbon dioxide in an unusual range of temperatures, pressure condition, pH and metal toxicity. So, organisms from the environment could be used for engineering microbes to solve the various technology options for carbon capture and storage.
